# Seroprevalence of Brucellosis in Livestock and Farmers’ Choice of Milk Marketing Channels in Rwamagana District, Rwanda

**DOI:** 10.4269/ajtmh.24-0410

**Published:** 2024-12-24

**Authors:** David Kiiza, Thomas Denagamage, Greg Kiker, Fiona Maunsell, Renata Serra, Lacey N. Harris-Coble, Bibiana Benavides, Jorge A. Hernandez

**Affiliations:** ^1^ Department of Large Animal Clinical Sciences, College of Veterinary Medicine, University of Florida, Gainesville, Florida;; ^2^ Department of Agricultural and Biological Engineering, University of Florida, Gainesville, Florida;; ^3^ Center for African Studies, University of Florida, Gainesville, Florida;; ^4^ Department of Geography, College of Liberal Arts and Sciences, University of Florida, Gainesville, Florida;; ^5^ Department of Animal Health, University of Nariño, Pasto, Colombia

## Abstract

Brucellosis remains an endemic disease in livestock populations in Rwanda, but the prevalence of the disease varies by geographic region. The common use of informal milk marketing channels represents a health hazard to humans when milk from *Brucella*-infected cows from one or more households is mixed with milk from other households for human consumption. In Rwanda, knowledge about the burden of brucellosis in livestock and factors associated with farmers’ choice of milk marketing channels is very limited. The objectives of this study were 1) to estimate the seroprevalence of brucellosis in livestock in Rwamagana District in Rwanda and 2) to estimate the frequency of and identify determinants associated with farmers’ choice of milk marketing channels. The seroprevalence of brucellosis in livestock at the household level was 1/160 (0.6%; 95% CI = 0.1–3.4%). Among 100 households that sold milk, 72 used informal milk trade channels. By the use of logistic regression, milk price and distance to closest milk collection center were positively associated with the use of informal milk marketing channels. Although the seroprevalence of brucellosis in livestock in Rwamagana District was low, the risk of disease transmission to humans through consumption of unpasteurized milk or milk products is not negligible. In this report, we present several policy options available to animal health authorities that can mitigate the risk of brucellosis disease transmission in populations.

## INTRODUCTION

Brucellosis remains an endemic disease in livestock populations in Rwanda. In livestock, *Brucella* infection can cause abortion and reduced milk yield.^1^^–^^3^ *Brucella* infection can be transmitted to humans through the consumption of unpasteurized milk or milk products from an infected animal or by direct contact with infected animals, particularly aborted fetuses.^4^ In humans, *Brucella* infection can cause fever combined with chills, headaches, back pain, loss of appetite, or fatigue.^5^^–^^7^ The socioeconomic consequences of brucellosis include forced unemployment in adults, school absenteeism in children, cost of treatment, and reduced livestock-related household income and access to milk, as well as potential trade restrictions of animals and animal products.^8^^–^^10^

In Rwanda, knowledge about the seroprevalence of brucellosis in livestock at the herd level is limited to three studies and varies widely based on management systems and geographical location. In one study, the seroprevalence of brucellosis was lower (5–12%) in households with dairy cattle raised under zero-grazing farm system conditions in Nyanza, Gicumbi, and Rwamagana Districts than in those in open-grazing farm system conditions in Nyabihu and Nyagatare Districts (24–52%).^11^ In a second study.^3^ the seroprevalence of brucellosis was lower (7%) in Muhanga District than in Nyabihu and Nyagatare Districts (42–89%). In Nyagatare District, most cattle herds are exposed to known risk factors associated with brucellosis (e.g., large herd size, use of shared water points, or exposure to infected or potentially infected wild animals).^3^^,^^11^ In that study,^3^ households were not randomly selected; thus, it is possible that the seroprevalence of brucellosis was under- or overestimated. In a third study,^12^ the herd seroprevalence of brucellosis in livestock in six districts (Gasabo, Gatsibo, Kayonsa, Musanze, Nyabihu, and Nyagatare) was 29%, but the herd prevalence by district was not reported. In all studies,^3^^,^^11^^,^^12^ sheep and goats (which are susceptible to *Brucella* infection) were not included.

It is estimated that 63–85% of the total milk produced in Rwanda (about 1 billion liters) is channeled through informal marketing channels, where milk is sold to milk traders, local restaurants, or households instead of milk collection centers (MCCs).^13^ In informal marketing channels, milk is transported using motorbikes, cars, or bicycles to Kigali City and other destinations, including kiosks, milk bars, restaurants, hotels, and households. In formal marketing channels, milk is transported from farms to MCCs, where the milk is tested and chilled and then transported to milk processing plants. Informal trade and consumption of raw milk are a health hazard in human populations, including *Brucella* infection, as milk from households with infected cows is mixed with milk from other households with infected or noninfected cows for human consumption. In 2016, Rwanda’s Ministry of Agriculture and Animal Resources (MINAGRI) published a Ministerial Order (MO) with guidelines for collection, transportation, and selling of milk to regulate milk market channels, improve milk quality, and prevent antimicrobial resistance and transmission of zoonotic diseases (i.e., brucellosis from livestock to humans),^13^ but farmer compliance is low. In one study, 82/96 (85%) farmers in Nyahibu District were engaged in informal milk marketing channels (e.g., sold milk to milk traders, local restaurants, or households instead of MCCs).^14^ In a second study, 270/413 (65%) farmers in Nyabihu District (Western Province) and Ruhango District (Southern Province) were engaged in informal milk marketing channels.^15^ Milk price had a positive effect on farmers’ choice of MCCs, but season, geographic location, and distance to MCCs can be potential confounding factors for the observed association between milk price and farmers’ choice of milk marketing channels. To our knowledge, no other studies have investigated determinants associated with farmers’ choice of milk marketing channels in Rwanda.

The objectives of the study are 1) to estimate the seroprevalence of brucellosis in livestock in Rwamagana District (Eastern Province) in Rwanda and 2) to estimate the frequency of and identify determinants associated with farmers’ choice of milk marketing channels, particularly milk price and distance to MCCs.

## MATERIALS AND METHODS

### Study site.

The study was conducted in Rwamagana District, Rwanda, from October 13 to December 5, 2023. Rwamagana is one of the seven districts that consititute Eastern Province in Rwanda, about 50 km from the capital city of Kigali. Rwamagana District has 14 sectors (Muhazi, Gishali, Kigabiro, Rubona, Munyaga, Munyiginya, Mwulire, Musha, Fumbwe, Gahengeri, Muyumbu, Karenge, Nzige, Nyakariro). The average annual rainfall is about 800 mm, and the rainy season is from September to December and from March to May. The District has six MCCs, in Kigabiro, Muhazi, Gishali, Rubona, Gahengeri (owned by farmer cooperatives), and Nzige (privately owned), approved by Rwanda’s MINAGRI. Milk is transported to Kigali through formal or informal milk marketing channels. The current milk price paid at MCCs is 300 Rwandan francs (Rwf) per liter.^16^

The criteria for selection of Rwamagana District were based on proximity to Kigali City and Rwanda’s Dairy Development Project selection criteria:^17^ 1) current level of cattle population and milk production; 2) current and projected market development potential, including investments in MCCs, dairy processing plants, animal feed factories, and evolving domestic and export market linkages; and 3) level of poverty, food insecurity, and malnutrition.

### Study design.

The study was designed as a cross-sectional study. Rwamagana District has a total of 19,226 households or farms with 1 to 210 cattle per household; most households (18,994 or 98.8%) have 1 to 10 cattle per household. A sample of 160 households with livestock was randomly selected from a study population of 18,994 households with 1 to 10 cattle per household from all 14 sectors that constitute Rwamagana District by using computer software (Research Randomizer; www.randomizer.org) and in coordination with the Rwanda Agriculture and Animal Resources Development Board (RAB). A sample size of 160 households with livestock (cattle, sheep, and goats) was justified using the following assumptions: study population = 18,994 households with livestock; expected herd seroprevalence of brucellosis = 12% ± 5%; confidence = 95%. The seroprevalence of 12% was justified based on a previously published study of brucellosis in cattle in Rwamagana District.^11^ Households with 1 to 10 adult cattle only or households with 1 to 10 adult cattle and sheep and goats were included. Because of funding limitations, households with more than 10 cattle (1.2%) were excluded. In addition, households with young cattle only, with bulls only, or with sheep and goats only were excluded.

### Outcomes.

Households with livestock classified as seropositive for brucellosis were those with one or more animals that tested positive for *Brucella* antibodies by using the Rose Bengal test (RBT) and ELISA.

Households with livestock engaged in formal trade of milk were those that sold milk to MCCs, and those with livestock engaged in informal trade of milk were those that sold milk to milk brokers, kiosks, milk bars, restaurants, hotels, or households.

### Collection of blood samples.

Within each study household with livestock, all cattle, sheep, and goats on the premises were sampled for detection of antibodies to *Brucella* spp. Blood samples (3–5 mL) were collected from the tail vein (coccygeal vein) in cattle or the jugular vein in sheep and goats. Red-top Vacutainer tubes without anticoagulant were used for the collection of blood samples. Each Vacutainer tube containing blood sample was labeled (animal identification number, household’s name and location, and date of blood sampling), placed in cool boxes, and transported the same day to the National Veterinary Laboratory in Kigali. Samples were assigned a unique laboratory identification number and centrifuged. Aliquots with blood serum were properly labeled and kept frozen at –20°C until further processing.

### Detection of *Brucella* antibodies.

Serum samples were tested for detection of antibodies to *Brucella* spp. by using the Rose Bengal test (RBT) and ELISA. Laboratory tests were conducted at the National Veterinary Laboratory of Rwanda in Kigali City. The reported sensitivity and specificity of the RBT were 100% and 100%, respectively,^18^ and for ELISA, these values were 100% and 99.74%, respectively.^19^

### Data collection.

The following data were collected from each study household: numbers of sheep, goats, female calves, male calves, heifers, dry cows, milk cows, and bulls on the premises, herd size, livestock system (zero-grazing, semi-intensive); number of cows (1, 2+); being part of a cooperative (yes, no); sells milk (yes, no), and if yes, to whom (MCC, village collector, milk shops, other households, other); average milk price per liter (Rwf); distance (in meters) to closest MCC; farmer knows what brucellosis is (yes, no); farmer identified abortion as the main syndrome of brucellosis (yes, no); cows are vaccinated against *Brucella* infection (yes, no, don’t know); history of abortion in the last 12 months (no, yes); cow breeding (artificial insemination, own bull, neighbor’s bull, artificial insemination or neighbor’s bull); and cattle introduction in the last 12 months (no, yes). The continuous variables for milk price and distance to the closest MCC were categorized into three or two groups based on 33rd or 33rd and 67th percentile distributions, respectively.

The geodesic distance from study households to the closest MCCs was calculated by using GPS coordinates for household and MCC that were collected using a handheld GPS device (Garmin Montana 700) and ArcGIS Pro software.^20^

## STATISTICAL ANALYSES

The prevalence of brucellosis in livestock at the household level was calculated by dividing the number of positive households by the total number of households tested (*n* = 160).^3^ Nine-five percent CIs were calculated for the prevalence estimate using software (Epitools, 2015). Among households that sold milk (*n* = 100), the proportion of households engaged in informal trade of milk was calculated by dividing the number of households engaged in informal trade of milk by the total number of investigated households. Nine-five percent CIs were calculated for the proportion estimate using the software.^21^

Descriptive statistics (number of households, median, minimum, and maximum) were calculated for continuous variables, including number of sheep, number of goats, number of female calves, number of male calves, number of heifers, number of dry cows, number of lactating cows, number of bulls, cattle herd size, herd size for total livestock (cattle, sheep, and goats), milk price per liter, milk yield per lactating cow, distance to closest MCC, calving interval, and lactation length. Comparison of households with livestock engaged in informal or formal trade of milk with continuous variables was conducted using a nonparametric method called the Wilcoxon rank sum test, and dichotomous variables were compared using the χ^2^ test.^22^

Logistic regression was used to identify investigated exposure factors (determinants) associated with study households engaged in informal trade of milk.^23^ Investigated exposure factors included livestock system, herd size, number of cows, being part of a cooperative, average milk price, distance from a study household to the closest MCC, farmer knows what brucellosis is, farmer identified abortion as the main syndrome of brucellosis, cows are vaccinated against *Brucella* infection, history of abortion in the last 12 months, cow breeding, and cattle introduction in the last 12 months. The variable for distance from the study household to the closest MCC or cooperative membership was forced in the final models to control for potential confounding effects on observed associations between investigated exposure variables (e.g., milk price) and informal trade of milk. A change in the odds ratio (OR) by ≥20% was considered evidence of confounding. Values of *P* <0.05 were considered statistically significant.^22^ The model goodness of fit for logistic regression was assessed using the Hosmer-Lemeshow test. The calculated ORs and their 95% CIs were used to measure the magnitude of association between investigated exposure factors and households engaged in informal milk marketing channels.

## RESULTS

**Figure 1.**
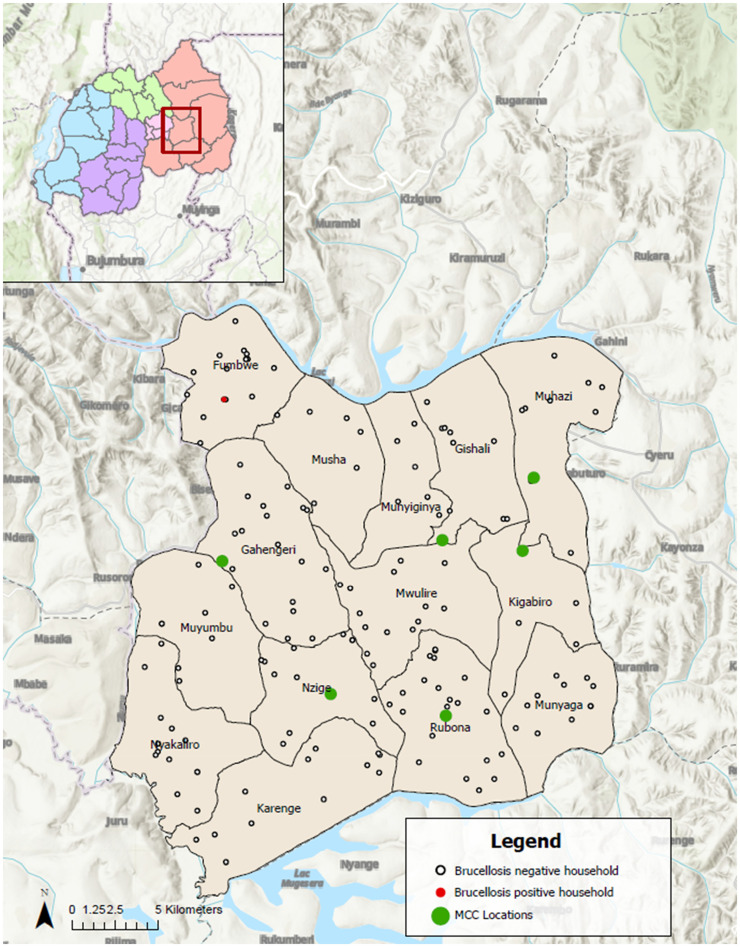
Map of Rwanda including Rwamagana District (red square) showing the locations of 160 study households with livestock and six milk collection centers (MCC).

The geographic locations of the 160 study households with livestock, a household with a cow that tested positive for *Brucella* antibodies, and six MCCs are presented in Figure 1.

**Figure 2.**
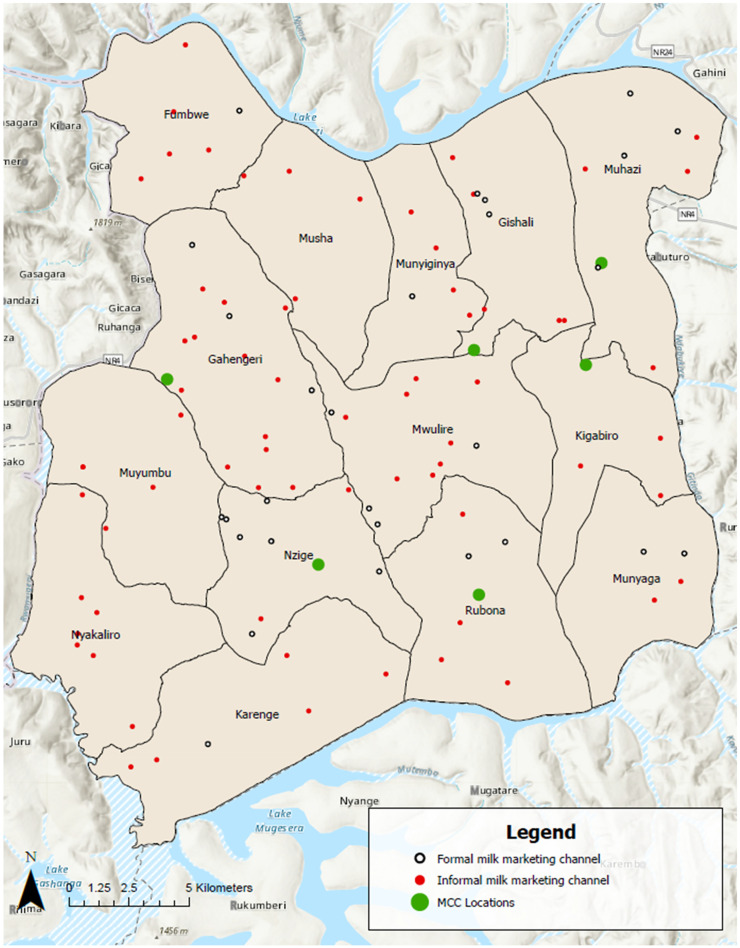
Map of Rwamagana District showing the locations of 100 study households with livestock that sold milk and were engaged in formal or informal milk trade channels and six milk collection centers (MCC).

The geographic locations of the 100 study households with livestock that sold milk and were engaged in either formal or informal milk trade channels and of the six MCCs are presented in Figure 2.

Among all 160 households with livestock, the median distance to the closest MCC was 4,726 m (118–13,898) (Table 1). Five of 160 (3%) households had > 6 cattle. In 11 (7%) households, the farmer reported that cattle were vaccinated against *Brucella* infection. Among 100 households that sold milk, the median milk price per liter was 300 Rwf (150–500).

**Table 1:** Description of 160 study households with livestock

Variable	Households, *n*	Median (minimum, maximum)
Sheep	160	0 (0, 6)
Goats	160	1 (0, 3)
Female calves	160	0 (0, 2)
Male calves	160	0 (0, 2)
Heifers	160	0 (0, 5)
Dry cows	160	0 (0, 3)
Milk cows	160	1 (0, 6)
Bulls	160	0 (0, 1)
Herd size (cattle, sheep, and goats)	160	4 (1, 21)
Herd size (cattle only)	160	3 (1, 11)
Average milk price per liter (Rwf)	100	300 (150, 500)
Distance to closest MCC (m)	160	4,726 (118, 13,898)
Average calving interval (months)	116	15 (12, 30)
Average milk lactation length (days)	117	330 (90, 750)
Average milk yield per day (liters)	122	7 (1, 36)

MCC = milk collection center; Rwf = Rwandan francs.

### Seroprevalence of brucellosis.

The seroprevalence of brucellosis at the household level was 1/160 or 0.6% (95% CI = 0.1–3.4%). In the one household that tested seropositive, 1 of 21 (4.8%) animals tested positive for *Brucella* antibodies. The household that tested positive was a semi-intensive production unit with 8 cattle and 13 goats; 1 of 8 cattle (a 6-year-old cow) tested positive for *Brucella* antibodies. The positive household was engaged in informal trade of milk (e.g., sold milk to other households for 400 Rwf per liter) and was 9,340 m away from the closest MCC.

In a population of about 19,000 households with livestock, the observed seroprevalence of brucellosis (0.6%) indicates that the number of households with one or more animals exposed to or infected with *Brucella* spp. is 114 in Rwamagana District.

### Farmers’ choice of milk marketing channels.

Among 100 households with livestock that sold milk, 72% (95% CI = 63–80%) were classified as being engaged in informal trade of milk. Among the 72 households, 40 (56%) sold milk to a village collector, 22 (30%) to other households, and 10 (14%) to milk shops.

The milk price was higher in households engaged in informal trade of milk (median = 300 Rwf) than in those engaged in formal trade of milk (median = 280 Rwf) (*P* = 0.004) (Table 2).

**Table 2:** Comparisons of households with livestock engaged in formal or informal trade of milk

Variable*	Value^†^ for Households Engaged in		*P*-Value
	Formal Trade (*n* = 28)	Informal Trade (*n* = 72)	
Sheep	0 (0, 3)	0 (0, 5)	0.659
Goats	1 (0, 12)	0 (0, 13)	0.137
Female calves	1 (0, 2)	0 (0, 2)	0.066
Male calves	0 (0, 2)	0 (0, 2)	0.193
Heifers	0 (0, 5)	0 (0, 3)	0.941
Dry cows	0 (0, 1)	0 (0, 3)	0.865
Milk cows	1 (0, 6)	1 (0, 4)	0.839
Bulls	0 (0, 0)	0 (0, 1)	0.090
Herd size (cattle, sheep, and goats)	5 (1, 20)	4 (1, 21)	0.304
Herd size (cattle only)	3 (1, 11)	3 (1, 9)	0.694
Average milk price per liter (Rwf)	280 (150, 400) (*n* = 27)	300 (200, 500)	0.004
Distance to closest MCC (m)	4,377 (238, 11,550)	4,887 (238, 13,898)	0.431
Average calving interval (months)	16 (12, 27) (*n* = 25)	15 (12, 30) (*n* = 58)	0.509
Average milk lactation length (days)	390 (180, 750) (*n* = 25)	330 (180, 720) (*n* = 59)	0.309
Average milk yield per day (liters)	8 (1, 36) (*n* = 26)	6.5 (1, 27)	0.158
Vaccination			
Yes	3 (11)	6 (8)	0.707
No or don’t know	25 (89)	66 (92)	
Cooperative membership			
No	21 (75%)	62 (86%)	0.184
Yes	7 (25%)	10 (14%)	

MCC = milk collection center; Rwf = Rwandan francs.

* Data are reported as median (minimum, maximum) unless indicated otherwise.

^†^ For variables where the sample size is less than 28 (formal trade) or less than 72 (informal trade), the actual sample size is reported.

By using variable logistic regression analysis, the variables for milk price and cooperative membership had *P*-values of <0.20 and were further examined (Table 3).

**Table 3:** Associations between investigated exposure factors and formal or informal trade of milk

Variable	Category	No. (%) of Households Engaged in		OR	95% CI	*P*-Value
		Formal Trade (*n* = 28 [100%])	Informal Trade (*n* = 72 [100%])			
Livestock system	Zero grazing	28 (100)	67 (93)	1.00	Reference	NA
	Semi-intensive	0 (0)	5 (7)	ND	ND	ND
Herd size (cattle, sheep, goats)	1–3	12 (43)	38 (53)	1.00	Reference	ND
	4–21	16 (57)	34 (47)	0.67	0.28–1.62	0.373
Cows*	1	17 (61)	46 (64)	1.00	Reference	NA
	2–8	11 (39)	26 (36)	0.87	0.36–2.14	0.767
Cooperative membership	Yes	21 (75)	62 (86)	1.00	Reference	NA
	No	7 (25)	10 (14)	0.48	0.16–1.43	0.189
Average milk price per liter (Rwf)	150–280	14 (52)	13 (18)	1.00	Reference	NA
	300	11 (41)	47 (65)	4.60	1.69–12.51	0.002
	320–500	2 (7)	12 (17)	6.46	1.21–34.53	0.029
Distance to MCC (m)	118–3,837	11 (39)	22 (30)	1.00	Reference	NA
	3,838–6,251	10 (36)	27 (38)	1.35	0.48–3.76	0.565
	6,252–13,899	7 (25)	23 (32)	1.64	0.54–5.00	0.381
Farmer knows what brucellosis is	Yes	7 (25)	11 (15)	1.00	Reference	NA
	No	21 (75)	61 (85)	1.85	0.63–5.38	0.260
Farmer identified the main syndrome of brucellosis as abortion	Yes	3 (11)	13 (18)	1.00	Reference	NA
	Other or don’t know	25 (89)	59 (82)	0.54	0.14–2.08	0.373
History of abortion in last 12 months	No	24 (86)	59 (84)	1.00	Reference	NA
	Yes	4 (14)	11 (16)	1.12	0.32–3.86	0.859
Cow breeding	AI	17 (61)	39 (57)	1.00	Reference	NA
	Own bull	0	3	ND	ND	ND
	Neighbor’s bull	7 (25)	19 (28)	1.18	0.42–3.33	0.750
	AI + neighbor’s bull	4 (86)	10 (15)	1.09	0.30–3.96	0.896
Cattle introduction in last 12 months	No	23 (82)	52 (72)	1.00	Reference	ND
	Yes	5 (18)	20 (28)	1.77	0.59–5.29	0.307
Vaccination against *Brucella* infection in cattle	Yes	3 (11)	6 (8)	1.00	Reference	NA
	No or don’t know+	25 (89)	66 (92)	1.32	0.31–5.68	0.709

AI = artificial insemination; MCC = milk collection center; NA = not applicable; ND = not determined; OR = odds ratio; Rwf = Rwandan francs.

* “Cows” includes heifers, dry cows, and milk cows.

The variables for milk price, cooperative membership, and distance to closest MCC were not correlated (*P* ≥0.327) with each other. The odds of informal trade were higher in households that sold milk for 300 Rwf (crude OR = 4.60; 95% CI = 1.69–12.51; *P* = 0.002) or 320–500 Rwf per liter (OR = 6.46; 95% CI = 1.21–34.53; *P* = 0.029) than in those that sold milk for ≤280 Rwf per liter. The odds of informal trade of milk was two times lower in cooperative members (OR = 0.48) than in nonmembers, but this association was not significant (*P* = 0.189).

In the multivariable analysis, informal trade of milk was positively associated with milk price (Table 4, model 1). The odds of informal trade were higher in households that sold milk for 300 RWF per liter (adjusted OR = 6.40; 95% CI = 2.11–19.46; *P* = 0.001) or 320–500 RWF per liter (adjusted OR = 8.99; 95% CI = 1.55–52.06; *P* = 0.014) after controlling for distance to the closest MCC (model 1). Adding the variable for distance to the closest MCC to the final model changed the crude ORs from 4.60 to 6.40 (39% change) and from 6.46 to 8.99 (39% change), an indication that the observed association between milk price and informal trade of milk was confounded (underestimated) by distance to the closest MCC. The odds of informal trade were 3.7 times higher in households that were ≥6,252 m away from the closest MCC than in those that were ≤3,837 m away (adjusted OR = 3.70; 95% CI = 0.97–14.16; *P* = 0.055), after controlling for milk price. The Hosmer-Lemeshow goodness-of-fit test (3.71; df = 4; *P* = 0.45) indicated that there is no evidence of poor fit for the data.

**Table 4:** Association between average milk price and informal trade of milk, after controlling for distance to closest MCC

Variable*	Category	Crude OR	Adjusted OR	95% CI	*P*-Value
Model 1					
Average milk price per liter (Rwf)	150–280	1.00	–	–	–
	300	4.60	6.40	2.11–19.46	0.001
	320–500	6.46	8.99	1.55–52.06	0.014
Distance to closest MCC (m)	118–3,837	1.00	–	–	–
	3,838–6,251	1.35	2.00	0.63–6.33	0.237
	6,252–13,899	1.64	3.70	0.97–14,16	0.055
Model 2					
Average milk price per liter (Rwf)	150–280	1.00	–	–	–
	300	4.60	4.98	1.79–13.88	0.002
	320–500	6.46	8.11	1.42–46.47	0.018
Cooperative membership	No	1.00	–	–	–
	Yes	0.40	0.40	0.12, 1.39	0.150

MCC = milk collection center; Rwf = Rwandan francs.

* Model 1: Hosmer-Lemeshow statistic = 3.71; *P* = 0.45; df = 4; Model 2: Hosmer-Lemeshow statistic = 2.29; *P* = 0.32; df = 2.

Finally, the odds of informal trade were higher in households that sold milk for 300 Rwf or 320–500 Rwf per liter after controlling for cooperative membership (Table 4, model 2). Adding the variable for cooperative membership to the final model changed the crude ORs from 4.60 to 4.98 (8% change) and from 6.46 to 8.11 (26% change), an indication that the observed association between milk price and informal trade of milk was confounded (underestimated) by cooperative membership. The association between cooperative membership and milk trade channels was not significant (*P* = 0.150) after controlling for milk price. The Hosmer-Lemeshow goodness-of-fit test (2.29; df = 2; *P* = 0.32) indicated that there is no evidence of a poor fit for the data.

## DISCUSSION

This study provides new information on the seroprevalence of brucellosis in livestock (cattle, sheep, and goats) and determinants associated with informal trade of milk in study households with livestock in Rwamagana District, Rwanda. A probabilistic, simple random sampling approach was used to select 160 households with livestock to mitigate selection bias. Furthermore, distance from study households to closest MCCs was measured by using a handheld GPS device and software to mitigate exposure bias (e.g., inaccurate measurement of distance between households engaged in informal milk marketing channels and distance to closest MCC).

### Seroprevalence of brucellosis.

Although the seroprevalence of brucellosis at the household level was low, the risk of disease transmission to humans is not negligible because more than 100 households with livestock can be expected to be exposed to *Brucella* spp. in Rwamagana District and because the frequency of households that sell milk and are engaged in informal milk marketing channels is high. The low prevalence of brucellosis can be explained by the fact that the majority of households with livestock in Rwamagana District are managed using zero-grazing farm system practices and artificial insemination. In Rwanda, the herd size in households with livestock engaged in zero grazing is small (1–2 cows), and livestock are confined within household property limits. The risk of exposure or disease introduction is lower than in households with more susceptible animals on the premises.^3^^,^^11^^,^^12^ In this study, 1 of 160 households was classified as seropositive for brucellosis. The household of interest had livestock raised using semi-intensive farm system practices, and only one of eight cows was classified as seropositive; the remaining seven cows and 13 goats tested negative. At the household level, it is possible that the result is a true positive because it has two known risk factors associated with a positive diagnosis of brucellosis: semi-intensive farm system and large herd size.^3^^,^^11^ Although the specificity of the ELISA used is very high, it is possible that the cow that tested positive for *Brucella* antibodies is a false positive. Funding limitations and the scope of the present study (which was limited to measuring seroprevalence) prevented us from conducting a more robust investigation, including the use of bacteriology and polymerase chain reaction (PCR) methods to confirm the diagnosis of *Brucella* infection.

One previous study estimated the seroprevalence of brucellosis in cattle in Rwamagana District.^11^ In that study, bulk milk samples from 8 of 66 (12%) households with dairy cattle tested positive by using an ELISA with high specificity. All 66 study households were engaged in zero-grazing farm practices. It is difficult to compare the previous seroprevalence estimate of 12% with that of 0.6% in our study because the sampling methods used were different. For example, in our study, the frequency of households with >6 cattle was lower (3%) than that in a study by Djangwani et al. (41%).^11^ However, the frequency of 41% applied to all 330 households with cattle under zero-grazing or open-grazing farming practices in five districts and was not specific for Rwamagana District. In addition, in our study, the sample size was justified by using an expected proportion of households with one or more animals positive for *Brucella* antibodies (12% prevalence) as a key assumption. In the study by Djangwani et al.,^11^ the assumption used was a proportion (69%) of households with livestock in five districts, including Rwamagana. Finally, in the study by Djangwani et al.,^11^ the systematic random approach used (a sampling approach where the first household was randomly selected, the next household was skipped, and the next one was selected, with this process being repeated until the required sample size was reached) most likely did not target all households at risk of exposure to *Brucella* spp., because households away from the first selected household were inherently excluded, introducing potential selection bias.

### Farmers’ choice of milk marketing channels.

Among 100 households that sold milk, 72% (63%, 80%) were classified as being engaged in informal trade of milk. The high frequency of informal trade of milk is similar to that reported in a previous study conducted in Nyabihu and Ruhango Districts (270/413 or 65%; 95% CI = 61–70%)^15^ as well as one in Nyabihu District (82/96 or 85%; 95% CI = 77–91%)^14^ prior to year 2016, when MINAGRI’s MO^17^^,^^24^ on milk collection, transportation, and marketing was implemented.

High milk price was associated with households engaged in informal milk marketing channels, after controlling for distance to the closest MCC. In our study, the observed association between a higher milk price and informal milk marketing channels differs from study results of Habiyaremye et al.,^15^ where milk price had a positive effect on farmers’ choice for MCCs. One explanation can be that in our study, the milk price reported by farmers applied to that offered during the rainy season, when milk supply is higher but milk price is fixed^16^ and lower at MCCs than in informal milk marketing channels. In contrast, the study results by Habiyaremye et al.^15^ were adjusted for season (dry season, rainy season), but the confounding effect of season on the observed association between milk price and farmer’s choice of milk market channels was not quantified or reported. Furthermore, the study by Habiyareme et al.^15^ did not report the milk selling price paid at MCCs or in informal milk marketing channels, but an assumption was made that MCCs offer better prices based on studies conducted in India and Thailand. Finally, in our study, long distance to the closest MCC was associated with farmers’ choice of informal milk trade channels, after controlling for milk price. Long distance to MCCs can impact transportation costs, risk of milk spoilage and rejection and related income, limited access to trusted sources of animal health and food safety information, and farmers’ time they can use for other farming activities. Our study results support the observation by Habiraremye et al.^15^ that a longer distance to a MCC may influence farmers to choose informal milk trade channels.

This study had several limitations. First, although all 160 study households were randomly selected, 81 (51%) were replaced by the closest household with livestock in the village because the household had no cattle (*n* = 22), the household did not exist (*n* = 18), the farmer was absent (*n* = 16), the animal(s) had died (*n* = 12), were sold (*n* = 10), or relocated (*n* = 2), or the farmer declined to participate (*n* = 1). In our assessment, the risk of selection bias was very low or negligible because 98.8% of the households in Rwamagana have 1 to 10 heads of cattle. Second, the study sample of 160 households was limited to those with ≤10 cattle. Thus, it is possible that the seroprevalence of brucellosis was underestimated because households with >10 cattle were not included. Third, only one cow in one household tested positive for *Brucella* antibodies. Although the blood sample tested positive by both the RBT and ELISA, it is possible that the test result is a false positive. Limited funding and laboratory capacity prevented our ability to use a gold standard test (e.g., bacteriologic culture or PCR). Fourth, the assessment of farmers’ choice for milk marketing channels was limited to data collected during the rainy season, when the milk supply is higher and the milk price is lower at MCCs. Finally, study results reported here apply to households with livestock in Rwamagana District. The results cannot be extrapolated to other districts or provinces in Rwanda.

## CONCLUSION

Although the seroprevalence of brucellosis in livestock was low in households with <10 cattle in Rwamagana District during the study period, the risk of disease transmission to humans through consumption of unpasteurized milk or milk products is not negligible. An observed seroprevalence of 0.6% at the household level implies that there are about 100 households with livestock exposed to or infected with *Brucella* spp. in a population of about 19,000 households with livestock in Rwamagana. In addition, because most farmers in Rwamagana District sell milk through informal channels, where milk from infected and noninfected cows is mixed without testing for quality control, disease transmission could potentially reach larger human populations.

Policy options available to the Rwandan government can include 1) business as usual or 2) implementation of enhanced risk management measures for control and prevention of brucellosis in populations. A business-as-usual scenario would consist of a) a low vaccination rate (≤15%) of female calves against *Brucella* infection and b) passive surveillance, where veterinarians send blood samples from livestock to a designated laboratory for diagnosis of brucellosis but an animal health response is not implemented when there is evidence of *Brucella* exposure in livestock and humans in selected households or populations at risk. The consequences can include disease transmission in livestock within and between households, from livestock to humans, or through consumption of raw milk distributed in informal milk marketing channels, forced unemployment in adults, school absenteeism in children, and potential animal trade barriers by milk-importing countries in the region. Option 2 is to implement an enhanced surveillance system for early detection and risk management of brucellosis in livestock by selecting one or more surveillance streams: a) syndromic surveillance, where a farmer reports an abortion in livestock to government veterinary services, an action that would trigger a field investigation by government veterinary services to confirm *Brucella* exposure or infection, identify the source of infection, and assess and mitigate potential disease transmission to other households with livestock in the village, cell, sector, or district; b) use MCCs as a surveillance stream for early detection and risk management of *Brucella* infection in livestock; or c) use herd testing but limited to households with >10 cattle. Additional policy options can include standard disease risk management measures in formally established brucellosis control programs. Examples include the following: 3) isolation of households with one or more animals that test seropositive for *Brucella* antibodies by using both the RBT and ELISA; 4) quarantine of exposed household/herd (e.g., direct or indirect contact with the positive household/herd); 5) an enhanced vaccination program, where all (100%) female calves are vaccinated against *Brucella* spp.; 6) enhanced education and communication; and 7) elimination of *Brucella*-infected animals. Animal health authorities can assess and decide what is the best course of action: business as usual or a selected option(s) above that is considered feasible and acceptable. The implementation of one or more enhanced risk management measures identified above for control and prevention of brucellosis in livestock populations would require the allocation of resources, a process that can be better justified by economic assessments to quantify their cost effectiveness.

Rwanda’s MO issued by the MINAGRI with guidelines for collection, transportation, and selling of milk is an important policy designed to regulate milk market channels and improve milk quality and prevent transmission of zoonotic diseases from livestock to humans (*Brucella* infection) through consumption of unpasteurized milk or milk products. Policy options can include 1) business as usual, where compliance is low in part because the milk price paid at MCCs is lower than that in informal milk marketing channels and because the number of MCCs is not adequate to cover the demand for milk collection, storage, and distribution, or 2) implementation of policy options already under consideration in Rwanda’s Dairy Development Project in Phase 2 at the national level. Examples of these policy options include 1) a payment system that recognizes and rewards the production of and marketing quality and safe milk^13^ and 2) the rehabilitation of MCCs and milk collection points and the construction of new MCCs and milk collection points.
